# The Importance of Intestinal Bacteria in Digestion

**Published:** 1899-09-23

**Authors:** 


### THE IMPORTANCE OF INTESTINAL BACTERIA IN DIGESTION.

The part played by micro-organisms in the human economy is not as yet fully understood. Thanks, however, to the wider views now prevailing on the subject of symbiosis—a biological phenomenon of extensive prevalence in the animal kingdom—a more comprehensive and consistent knowledge has gradually grown up concerning the *rôle* of micro-organisms normally present in the human body. Many species of bacteria are now known to be present in the alimentary canal, and of these some have their habitat in the stomach and others in the small intestine, while quite a host of bacteria and other fungi are found to inhabit the lower bowel in both man and the herbivorous animals. Ten years ago Dr. Vignal,* of Paris, published records of an important series of researches made by him on the characters and functions of micro-organisms found to be habitually present in the stomach and intestines of man. Specimens were taken either from the stomach directly, or from the intestines in case of intestinal fistula or of colotomy, &c., and pure cultures were made of the isolated microbes thus obtained. The action of these microbe cultures on raw or partially digested food stuffs was carefully tried under conditions of moisture, temperature, &c., resembling as nearly as possible those prevailing in the alimentary canal. A large number of experiments were made, and the results on the whole were found to be very concordant. Thus it appeared that certain microbes from the stomach had properties similar to those of saliva or starch, and aided the process of starch digestion (diastatic action), while others had the power (in an acid medium) of accelerating the digestion of meat and of albuminous substances (proteolytic action). Micro organisms from the jejunum were found to act best in a neutral or faintly alkaline medium, and some, again, had a slight action in aiding the digestive action of bile on fats. Suffice it to say that Dr. Vignal's experiments (which were extensively carried out) went far to show that microbes habitually present in the stomach and intestines played an important and in all probability a beneficial part in aiding the digestion of the food-stuffs in the alimentary canal. In a recent number of the *Archiv-fur Hygiene und Infektions Krankheiten*, XXXIV., 1899, Dr. M. Schottelius gives a record of experiments as to the importance and significance of intestinal bacteria in the nutrition and growth of chickens. For this purpose he made use of chickens hatched under the most careful and approved aseptic conditions. Though the plan is *à priori* simple, yet the apparatus and actual means employed were very complicated. Nevertheless, the experiments were successfully carried out, and the chickens lived and moved about and fed in sterilised vessels, breathed a sterilised air, and were fed on sterilised food. The experiments showed that chickens so brought up gained but little in size or weight compared with those brought up under ordinary conditions. Thus the maximum increase of weight in twelve days after hatching was 25 per cent. of body-weight, and after the twelfth day there was no increase in weight, but, if anything, a decrease. On the other hand, chickens brought up under ordinary conditions increased 140 per cent. by the twelfth day, and by the seventeenth day the increase of weight was on an average 250 per cent. It thus followed that in young chickens the bacteria present in the food and in the alimentary canal were useful in the digestion of food. Cultures were made of the excreta of the "protected" chickens as of the ordinary ones. Those prepared from the "protected" chickens showed no micro-organisms, and for the first four to eight days there was no diastase. Later on, however, diastase began to appear, and could be detected in the culture medium, but there were as yet no bacteria. In the excreta of chickens brought up in the ordinary way abundant bacteria were found, and the diastatic action was pronounced. These facts tended to show the utility of bacterial co-operation in aiding digestion, and this conclusion found additional confirmation in the fact that the excreta of all chickens, whether "protected" or not, were free from microbes during the first 36 or 40 hours; and it is to be noted that during this period the chickens, although they took food, yet scarcely increased in weight. With the presence of bacteria in the alimentary canal, however, the digestive processes went on apace, and the animals rapidly grew and gained weight.

* Archives de Physiologie, 1898-9.

